# Prognostic heterogeneity and clonal dynamics within distinct subgroups of myelodysplastic syndrome and acute myeloid leukemia with *TP53* disruptions

**DOI:** 10.1002/jha2.791

**Published:** 2023-09-11

**Authors:** Shyam A. Patel, Jan Cerny, William K. Gerber, Muthalagu Ramanathan, Asiri Ediriwickrema, Benjamin Tanenbaum, Lloyd Hutchinson, Xiuling Meng, Julie Flahive, Bruce Barton, Andrew J. Gillis‐Smith, Sakiko Suzuki, Salwa Khedr, William Selove, Anne W. Higgins, Patricia M. Miron, Karl Simin, Bruce Woda, Jonathan M. Gerber

**Affiliations:** ^1^ Division of Hematology and Oncology, Department of Medicine UMass Memorial Medical Center, UMass Chan Medical School Worcester Massachusetts United States; ^2^ Institute for Stem Cell Biology & Regenerative Medicine; Division of Hematology, Department of Medicine Stanford University Stanford California United States; ^3^ Department of Pathology UMass Memorial Medical Center, UMass Chan Medical School Worcester Massachusetts United States; ^4^ Department of Population & Quantitative Health Sciences UMass Chan Medical School Worcester Massachusetts United States; ^5^ Dept. of Molecular Cell & Cancer Biology UMass Chan Medical School Worcester Massachusetts United States

**Keywords:** TP53, myelodysplastic syndrome, acute myeloid leukemia, hematopoietic stem cell, clonal dynamics

## Abstract

*TP53* aberrations constitute the highest risk subset of myelodysplastic neoplasms (MDS) and acute myeloid leukemia (AML). The International Consensus Classification questions the blast threshold between MDS and AML. In this study, we assess the distinction between MDS and AML for 76 patients with *TP53* aberrations. We observed no significant differences between MDS and AML regarding *TP53* genomics. Median overall survival (OS) was 223 days for the entire group, but prognostic discrimination within subgroups showed the most inferior OS (46 days) for AML with multihit allelic state plus *TP53* variant allele frequency (VAF) > 50%. In multivariate analysis, unadjusted Cox models revealed the following variables as independent risk factors for mortality: AML (vs. MDS) (hazard ratio [HR]: 2.50, confidence interval [CI]: 1.4–4.4, *p* = 0.001), complex karyotype (HR: 3.00, CI: 1.4–6.1, *p* = 0.003), multihit status (HR: 2.30, CI 1.3–4.2, *p* = 0.005), and absence of hematopoietic cell transplant (HCT) (HR: 3.90, CI: 1.8–8.9, *p* = 0.0009). Clonal dynamic modeling showed a significant reduction in *TP53* VAF with front‐line hypomethylating agents. These findings clarify the impact of specific covariates on outcomes of *TP53*‐aberrant myeloid neoplasms, irrespective of the diagnosis of MDS versus AML, and may influence HCT decisions.

## 1 INTRODUCTION

A decade has elapsed since comprehensive sequencing efforts from The Cancer Genome Atlas revealed that *TP53* mutations are recurrent in acute myeloid leukemia (AML), with a mutational frequency of 8% [1]. AML and myelodysplastic neoplasms (MDS) harboring *TP53* mutations are associated with exceptionally poor survival, typically on the order of weeks to months [2, 3, 4, 5, 6, 7]. Specifically, very recent efforts that have clarified this genetically defined subgroup include the IPSS‐M model for MDS, the European LeukemiaNet (ELN) 2022 classification for AML, and the 2022 International Consensus Classification (ICC) [8, 9, 10]. The IPSS‐M model has shown an adjusted hazard ratio (HR) of 3.27 (the highest of all) for multihit *TP53* aberration; the ELN 2022 classification now includes a new category termed “AML with mutated *TP53*” for patients with *TP53* variant allele frequency (VAF) ≥ 10%; and the 2022 ICC has designated a unique category for MDS and AML with *TP53* mutation at the height of the myeloid neoplasm classification hierarchy.

Our group has recently explored the basis for these distinctly adverse outcomes in *TP53*‐aberrant MDS and AML using clonal dynamic modeling, which suggested that *TP53*‐aberrant clones drive malignant hematopoiesis and constitute measurable residual disease (MRD) for most patients, which in turn contributes to relapse [3]. Genomic aberration affecting the *TP53* locus is often associated with disruption of other cellular machinery including regulators of the epigenome, the spliceosome complex, and the cohesin complex [3]. However, our study size was relatively small, and our analysis was conducted under the assumption that all *TP53*‐mutant MDS and AML are equal (a homogeneous population). The population of patients with *TP53* disruptions is more heterogeneous than current classification and prognostication systems suggest, and data on prognostic discrimination within this heterogeneous population are limited. The distinction between *TP53*‐mutant MDS versus AML using the time‐honored World Health Organization (WHO) blast cutoff of 20% is arbitrary, and very recent studies have shown negligible differences in both genetic profiles and clinical outcomes between MDS and AML [11].

## 2 MATERIALS AND METHODS

### 2.1 Patient subjects

UMass Institutional Review Board approval was obtained via protocols H00019393 (“AML Database”) and H00013131 (“Blood & Marrow Banking”; NCT01174615), as previously described [3]. The UMass Leukemia Registry identified 76 patients harboring one or more aberrations in *TP53* plus with an ICD‐10 code of either D46.9 (MDS, including its subentities) or C92.00 (AML, including its subentities) between 2011 and 2023. *TP53* aberrations were defined as mutations within the *TP53* locus (missense, frameshift deletion, splice site deletion, in‐frame deletion, frameshift insertion) or large‐scale disruptions of 17p (deletion, addition, translocation, isochromosome, duplication, inversion, dicentric chromosome, ringed chromosome), confirmed by fluorescence in situ hybridization (FISH) to have *TP53* deletion.

### 2.2 Cytogenetic analyses

Bone marrow cells from patients with *TP53* aberrations were cultured, and metaphase cells were harvested at 24 and 48 h. For these 76 patients, conventional karyotyping by G‐banding using trypsin and Giemsa was performed on metaphases. For each specimen, at least 20 (or all available) metaphase cells were analyzed for numerical and structural chromosomal anomalies. Criteria defined by the International System for Human Cytogenetic Nomenclature were used to describe abnormal clones. FISH was performed for *TP53* deletion assessment using the Vysis LSI TP53/CEP17 probes (Abbott Molecular).

### 2.3 Profiling of TP53 somatic variants and allelic states

Genomic DNA was purified from bone marrow aspirates, as previously described [3]. A library was created using multiplex PCR targeting oncogene “hot spot” mutations and full exon sequencing (CTMPv3 panel). PCR amplicons were isolated, followed by the ligation of a sequencing linker, and a barcode for patient identification. Real‐time PCR was used to quantify the library. A sequencing template was prepared by diluting the library, and performing emulsion PCR to amplify and link the amplicon to a sequencing bead. The sequencing template was loaded onto an IonTorrent PGM next‐generation sequencer. For *TP53* exome sequencing, the entire coding region from codons 1–393 of isoform A was covered, with coverage depth ranging from 500× to 6500× (Supporting Information Table S1). Patients were stratified by the presence or absence of multihit status and by *TP53* VAF. Multihit status was defined as per the 2022 ICC: two or more distinct *TP53* mutations with VAF ≥ 10%, or a single *TP53* mutation plus one of the following abnormalities: (1) *TP53* deletion, (2) *TP53* VAF > 50%, (3) copy‐neutral loss‐of‐heterozygosity, or (4) any complex karyotype [10]. Monoallelic *TP53* status was defined as non‐multihit.

### 2.4 Clonal dynamic modeling and copy number variation (CNV) analysis

Clonal and subclonal structures for each patient with available serial bone marrow aspirates were modeled via R (R Core Team, 2013) and R packages “circlize,” “timescape,” and “BioCircos” as previously described [3, 12]. VAF for each mutant gene was adjusted for gene copy number variation (CNV) in the event of copy number gain or loss. VAF was defined as the ratio of mutant read number to total read number (total reads = mutant reads plus wild‐type reads). CNV was performed on sequencing data for samples containing ≥20% tumor load and analyzed on IonXpress. CNV annotation against VAF was under the assumption that, for a given bone marrow sample, mutations with VAFs in a similar range likely occur in the same clone.

### 2.5 Hierarchical clustering for VAFs for TP53 and cooperating mutations

Unsupervised hierarchical clustering of VAFs for *TP53* and co‐occurring mutations was performed by the R package “pheatmap.” Clades were generated based on type and VAF for co‐occurring mutations. VAF data were analyzed using IonTorrent Variant Caller and Softgenetics Next Gene software packages using GRCh37_3 (Ver HG19) as the reference sequence.

### 2.6 Multivariate analysis for survival

Cox proportional hazard models were used to analyze the binary dependent variable of interest (alive vs. deceased). HR and 95% confidence intervals were reported for each model. Variables were removed one by one from the adjusted model based on the statistical significance in the model. Age was kept in the model despite not being statistically significant in the adjusted model because of its clinical association with mortality. Analyses were conducted using SAS software (version 9.4; SAS Institute, Cary, NC). α was set as 0.05 for all tests.

### 2.7 Other statistical analyses

Pearson's chi‐squared test for significance was used to calculate observed vs. expected frequencies for sex and ethnicity distributions. A Paired student's *t*‐test assuming heteroscedasticity was performed to assess differences in fold changes in *TP53* VAF with treatment. Log‐rank tests (nonparametic) were performed to compare survival distributions of subsets of *TP53*‐aberrant MDS and AML.

## 3 RESULTS

### 3.1 Demographics and baseline features of *TP53*—Aberrant MDS versus AML

We queried the laboratory information system and the UMass Leukemia Registry for patients with a diagnosis of MDS or AML between 2011 and 2023. A total of 598 patients were identified. Of the 598 patients, 76 (12.7%) harbored *TP53* aberrations. Ten patients had disruption of the *TP53* locus without an intragenic mutation. We assessed baseline features based on morphologic classification into MDS and AML, as defined by the WHO Revised 4th Edition [13]. Of these 76 patients, 35 (46.1%) had MDS and 41 (53.9%) had AML. Of the 35 patients with MDS, 9 (25.7%) had therapy‐related MDS (the most prevalent subtype of MDS). Of the 41 patients with AML, 17 (41.5%) had AML, not otherwise specified (NOS) and 14 (34.1%) had AML with myelodysplasia‐related changes (AML‐MRC), which were the two most prevalent subtypes of AML (Table 1). The majority of patients with therapy‐related myeloid neoplasms or multihit states also had a complex karyotype.

**TABLE 1:** Baseline characteristics for MDS and AML with *TP53* disruption.

	MDS	AML
	No. of events/total no. (%)	
All patients (*n* = 76 total)	35/76 (46.1)	41/76 (53.9)
Age		
Median (range), year	76.2 (41.6–93.4)	67.3 (46.3–89.5)
Sex		
Female	14/35 (40.0)	16/41 (39.0)
Male	21/35 (60.0)	25/41 (61.0)
Ethnicity		
Non‐Hispanic White	31/35 (88.6)	37/41 (90.2)
Hispanic/Latinx	4/35 (11.4)	3/41 (7.3)
Black/African American	0/35 (0.0)	1/41 (2.4)
Asian (East Asia, South Asia)	0/35 (0.0)	0/35 (0.0)
WHO HAEM4R Subtype		
*t*‐MDS	9 (25.7)	
MDS‐EB‐1	7 (20.0)	
MDS‐EB‐2	6 (17.1)	
MDS‐MLD	5 (14.3)	
MDS‐SLD	4 (11.4)	
MDS with isolated del(5q)	2 (5.7)	
MDS, unclassifiable	1 (2.9)	
MDS/MPN, unclassifiable	1 (2.9)	
AML, NOS		17 (41.5)
AML‐MRC		14 (34.1)
*t*‐AML		8 (19.5)
Myeloid sarcoma		2 (4.9)
ECOG performance status		
0–1	55.6	51.9
2–3	44.4	48.1
Bone marrow blast		
Median (range), %	6 (0–17.5)	43 (0–93)
Baseline CBC–Median ± SE		
WBC (×10^3 cells/μL)	3.6 ± 1.0	3.6 ± 2.6
Hemoglobin (g/dL)	8.5 ± 0.3	8.5 ± 0.3
Platelets (×10^3 cells/μL)	73.0 ± 21.6	36.0 ± 11.9
ANC (×10^3 cells/μL)	1.4 ± 0.9	0.7 ± 0.5
Frequency of co‐mutations (%)	51.4	46.3
Front‐line therapy administered		
Cytarabine‐based	0 (0.0)	9 (22.0)
HMA‐based	26 (74.3)	27 (65.9)
None/supportive care	9 (25.7)	5 (12.2)

Abbreviations: ANC, absolute neutrophil count; CBC, complete blood count; EB, excess blasts; HMA, hypomethylating agent; MLD, multilineage dysplasia; MRC, myelodysplasia‐related changes; NOS, not otherwise specified; SLD, single‐lineage dysplasia.

The median age at diagnosis for MDS was significantly higher than that of AML: 76.2 years (range: 41.6–93.4) versus 67.3 years (range: 46.3–89.5), *p* = 0.0079 (Table 1). There was a nonuniform distribution between sexes; most were males (*p* = 0.0476). Ethnicity representation among patients with *TP53* disruptions was significantly different compared to that of the reference population from the 2020 Worcester County Census (*p* = 0.0289) (www.census.gov). Most patients were non‐Hispanic White. Patients with MDS and AML had no significant differences in median WBC and hemoglobin values at diagnosis, but patients with AML had lower median platelet count compared to those with MDS (Table 1).

### 3.2 Subtypes of *TP53* aberrations in MDS versus AML

**FIGURE 1:**
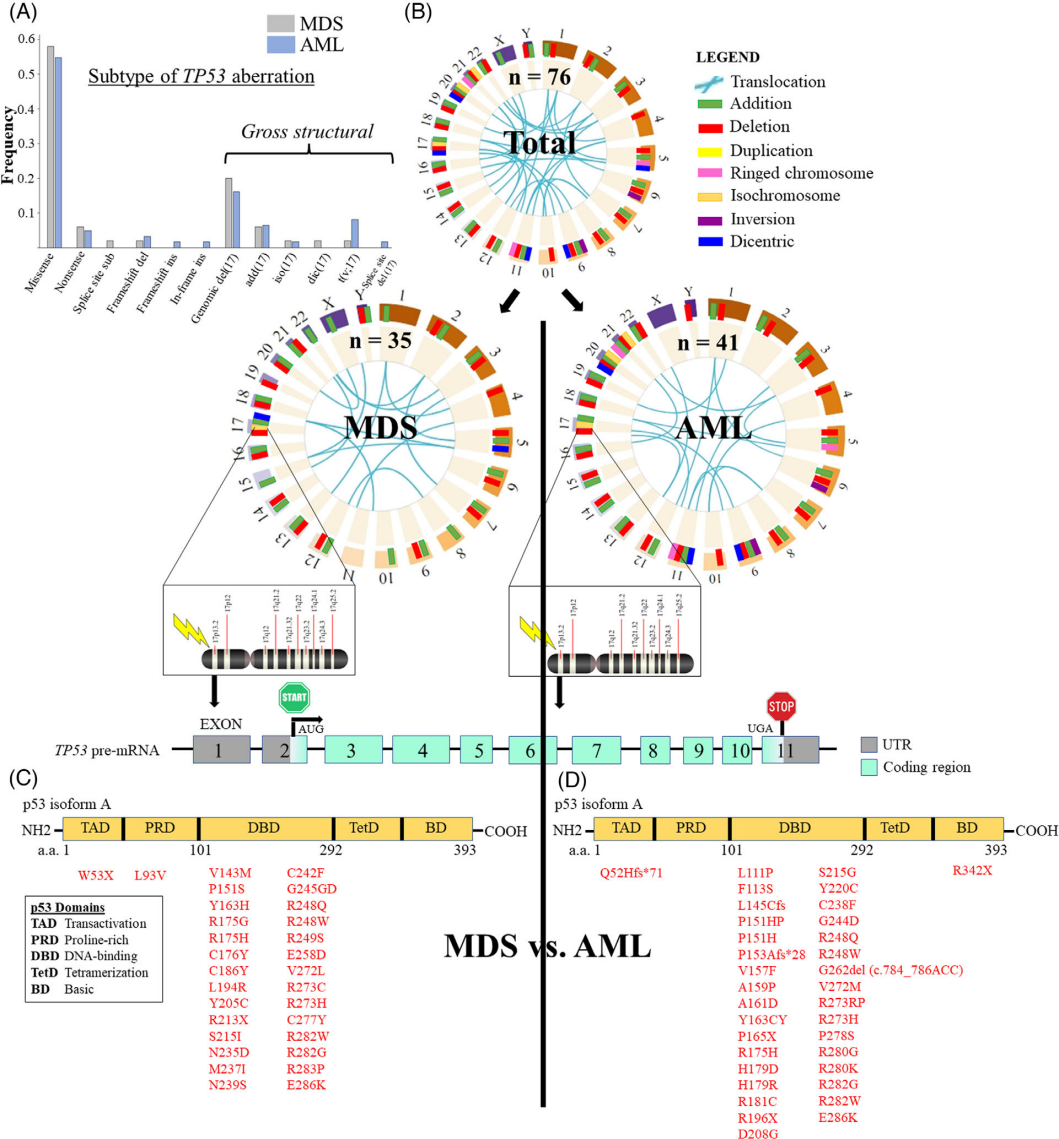
(A) Subtype and frequency of *TP53* aberrations found in 76 total patients with either MDS (*gray*) or AML (*blue*). Gross structural changes involving chromosome 17 are included. (B) Gross structural changes across all human chromosomes for both MDS and AML (*top chromomap*). Total annotation of structural changes was divided into MDS (*left chromomap*) or AML (*right chromomap*). Topology of intragenic *TP53* disruptions along p53 isoform A for MDS (C) or AML (D). Open reading frame encoding p53 isoform A is shown.

We assessed subtypes of *TP53* aberrations in WHO‐defined MDS vs. WHO‐defined AML for 76 patients with *TP53*‐aberrant MDS or AML. The most prevalent subtype of aberration was the missense mutation (Figure 1A). There were no significant differences in the frequencies of these aberrations between MDS and AML. We assessed all gross structural abnormalities found in patients with *TP53* aberrations, then segregated the abnormalities by MDS versus AML (Figure 1B). The structural genomic landscape from aggregate analysis did not appear different between MDS and AML: eight major subtypes of structural disruptions were observed in both MDS and AML, dispersed among nearly all chromosomes. We then assessed the topographic distribution of intragenic *TP53* disruptions in the open reading frame for *TP53*‐encoding p53 isoform A for MDS (Figure 1C) and AML (Figure 1D). Among 30 unique *TP53* intragenic mutations in patients with MDS, 28 (93.3%) localized to the DNA‐binding domain, with only 1 mutation (3.3%) each in the transactivation domain and the proline‐rich domain. Among 35 unique *TP53* intragenic mutations in patients with AML, 33 (94.3%) localized to the DNA‐binding domain, with only 1 mutation (2.9%) each in the transactivation domain and the basic domain.

### 3.3 Clonal combinations among varying *TP53* allelic states of MDS versus AML

**FIGURE 2:**
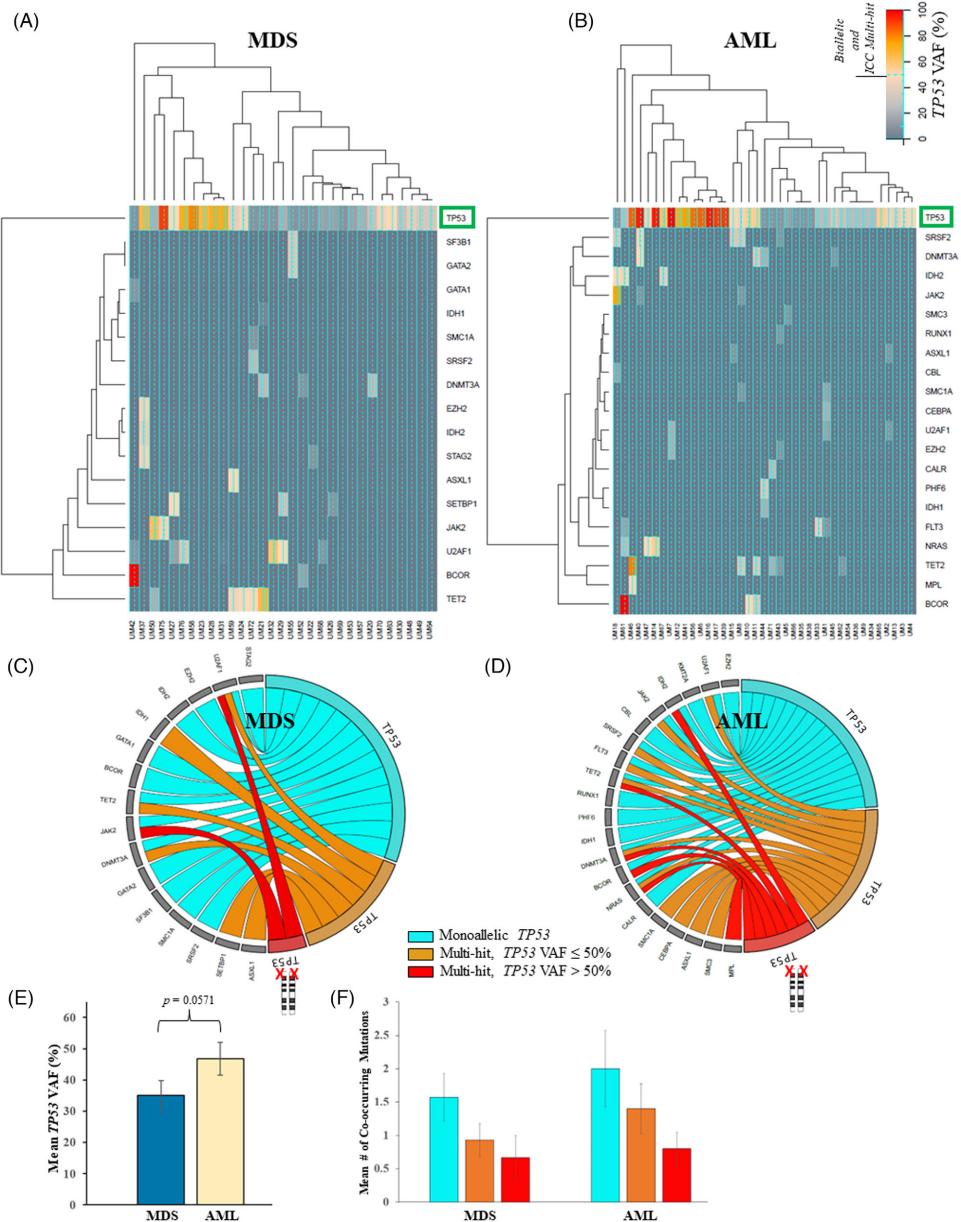
*TP53‐*centric oncoprint for MDS (A) versus AML (B). Unsupervised hierarchical clustering was performed based on VAFs for co‐occurring mutations from diagnostic bone marrow specimens for patients with *TP53* aberrations. Biallelic and ICC‐defined multihit state is demarcated in yellow and red in clustered heatmaps. Co‐occurring mutational profile stratified by *TP53* allelic state and VAF is shown for MDS (C) versus AML (D). Data represent patients with any subtype of *TP53* disruption (including loss of the *TP53* locus, not just intragenic *TP53* mutation). Width of chords does not correlate with frequency of co‐occurrence. (E) Mean *TP53* VAF on diagnostic bone marrow specimens for MDS versus AML. (F) Co‐occurring mutation frequency per patient as a function of *TP53* allelic state and VAF. Mean ± SE is shown.

We next assessed the co‐occurring mutational profiles for MDS versus AML with *TP53* aberrations based on VAFs for mutations from diagnostic bone marrow specimens (Figure 2A, B). Full exon sequencing was performed for known recurrent somatic mutations. There were 16 distinct co‐occurring mutations among patients with *TP53*‐aberrant MDS (Figure 2A) and 20 distinct co‐occurring mutations among patients with *TP53*‐aberrant AML (Figure 2B).

Unsupervised hierarchical clustering showed a largely dichotomous distribution with respect to co‐occurring mutational profiles for both MDS and AML (Figure 2A, B, *dendrograms*). The clades with high versus low *TP53* VAF showed no obvious differences in co‐occurring mutations, and there was no significant difference in the dendrograms of co‐occurring mutations for MDS versus AML. Therefore, we mapped co‐occurring mutational profiles as a function of *TP53* allelic state (monoallelic vs. multihit) and absolute VAF (≤50% or >50%) for MDS (Figure 2C) and AML (Figure 2D). The frequency of multihit status was higher for AML compared to MDS (73.2 vs. 60.0%), suggesting that multihit status correlates with more aggressive myeloid neoplasm. The frequency of patients with multihit status plus VAF >50% was also higher for AML compared to MDS (36.6 vs. 17.1%).

There was no significant difference in the mean *TP53* VAF for patients with AML (46.8 ± 5.3%) versus MDS (35.0 ± 4.8%) (*p* = 0.0571) (Figure 2E). There was no significant difference in the subclonal diversity for MDS versus AML (Figure 2F).

### 3.4 Prognostic discrimination within a heterogeneous population of *TP53*—aberrant MDS and AML

**FIGURE 3:**
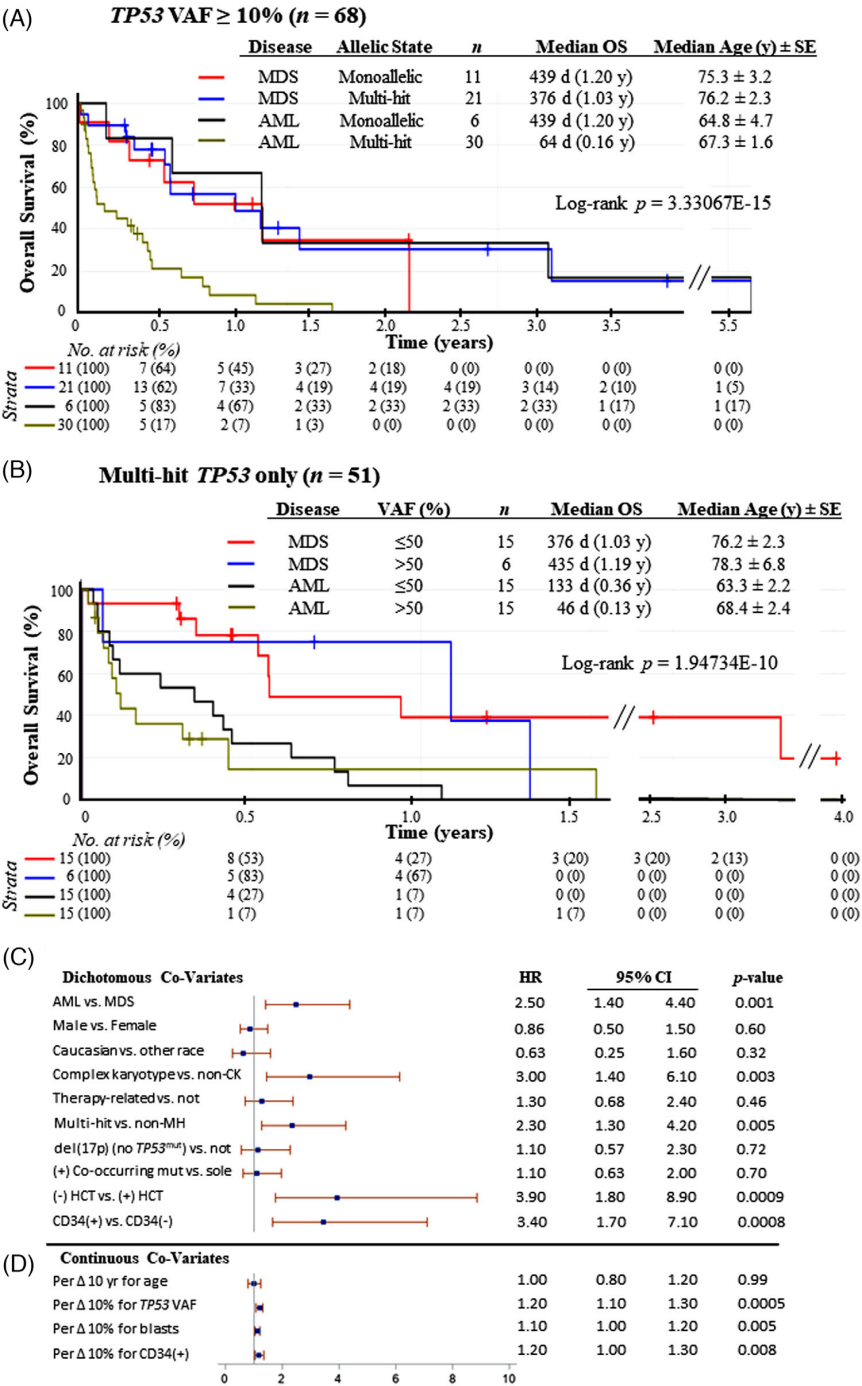
Overall survival for the heterogenous population of *TP53*‐aberrant MDS and AML. (A) All patients with *TP53* VAF ≥ 10% were included and stratified by clinical diagnosis (MDS vs. AML) and by allelic state. (B) Patients with multihit status only were analyzed and segregated by absolute *TP53* VAF (below or above 50%). Breaks in *x*‐axis are shown with double diagonal lines. Log‐rank *p* values for survival differences are shown. (C) Multivariate analysis using Cox proportional hazard model for dichotomous variables. (D) Multivariate analysis using Cox proportional hazard model for continuous variables :age, *TP53* VAF, blast percentage at diagnosis, and CD34^+^ cell percentage. *Legend*: HR, hazard ratio; CI, confidence interval; SE, standard error.

We next assessed overall survival (OS) for varying *TP53* allelic states and mutational burdens (VAFs) for MDS and AML. We identified 68 patients who met the 2022 ICC definition for myeloid neoplasm with mutated *TP53* (after exclusion of patients with VAF < 10%) and stratified patients based on hit status and VAF. Median follow‐up was 214 days (very short due to the deaths of patients). Median OS was not significantly different among patients with monoallelic *TP53*‐mutant MDS, multihit *TP53*‐mutant MDS, and monoallelic *TP53*‐mutant AML (median OS: 439 days vs. 376 days vs. 439 days, respectively) (Figure 3A). In contrast, patients with multihit *TP53*‐mutant AML had distinctly shorter survival (median: 64 days) (Figure 3A).

We then assessed OS for patients in the multihit subgroup only (*n* = 51) (Figure 3B). We substratified patients based on *TP53* VAF, given that VAF > 50% confers an obligatory biallelic disruption within the multihit subgroup. OS for patients with MDS with VAF ≤ 50% and VAF > 50% was not significantly different (median: 376 days vs. 435 days, respectively) (Figure 3B). Patients with AML with *TP53* VAF > 50% had significantly inferior OS (median: 46 days) (Figure 3B). OS was also analyzed for the entire cohort (Supporting Information Figure S1A) and also as a function of disease category (Supporting Information Figure S1B), karyotype (Supporting Information Figure S1C), and antecedent chemotherapy exposure (Supporting Information Figure S1D).

Unadjusted survival models showed significant associations with mortality in multivariate analysis (Figure 3C). Patients with AML had 2.5 times the risk of death as those with MDS. Death was predominantly due to disease progression. Patients with a complex karyotype were at 3 times the risk of death than those without it. Patients with multihit status were 2.3 times at risk of death as compared to those without. Those who did not receive hematopoietic stem cell transplant (HCT) were at 3.9 times the risk of death as compared to HCT recipients. The hazard was analyzed for the following continuous variables: age, *TP53* VAF, blast percentage at diagnosis, and CD34^+^ cell percentage (Figure 3D). For every absolute increase by 10% in *TP53* VAF, blast percentage at diagnosis, and CD34‐ cell percentage, the risk of death increased by 20, 10, and 20%, respectively. We also assessed OS as a function of *TP53* VAF as a continuous variable, regardless of the MDS versus AML designation, and noted an inverse correlation between OS and *TP53* VAF for patients with complex karyotypes (Supporting Information Figure S2A). There was also an inverse correlation between OS and age at diagnosis (Supporting Information Figure S2B).

The results from the adjusted model also showed an association between mortality and AML, HCT status, and change in *TP53* VAF (Supporting Information Table S2). Patients with AML had 3.3 times the risk of death as those with MDS while holding constant HCT status, *TP53* VAF, and age. Those without HCT were at 4.3 times the risk of death than HCT recipients, while holding constant AML and MDS status, *TP53* VAF, and age. For every absolute increase in *TP53* VAF by 10%, the risk of death increased by 20% while holding constant AML and MDS status, HCT status, and age (Figure 3D).

There was no difference in survival for *t*‐MN versus non‐*t*‐MN (Figure 3C and Supporting Information Figure S1D), suggesting that *TP53* aberration is a confounding variable with regard to the association between *t*‐MN and increased risk for death (for patients with *TP53*‐aberrant MDS or AML).

### 3.5 Clonal and subclonal dynamics as a function of therapeutic intervention

**FIGURE 4:**
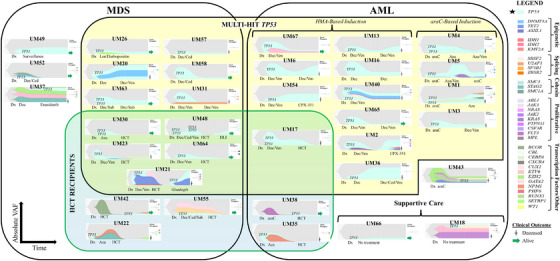
Dynamics of clonal response, organized by WHO top‐line diagnosis and *TP53* allelic state, as a function of therapeutic intervention. Clonal trajectories for all patients who underwent serial bone marrow evaluations during treatment. Timepoints are demarcated by clinical course, including diagnosis, intervention administered, HCT, etc. Patients with multihit *TP53* are shown in yellow backdrop, and HCT recipients are shown in green backdrop. Legend: araC, cytarabine‐based; Aza, azacitidine; Ced, cedazuridine; DLI, donor lymphocyte infusion; Dec, decitabine; Dx, diagnosis; HCT, hematopoietic cell transplant; Len, lenalidomide; Sab, sabatolimab; Ven, venetoclax.

Our prior studies suggested that inferior survival for patients with *TP53* disruptions was associated with the persistence of *TP53*‐mutant clones [3]. However, we previously did not discern clonal dynamics between disease designations (MDS vs. AML) or *TP53* allelic states. We therefore modeled the clonal dynamics of heterogeneous subsets of *TP53*‐aberrant MDS versus AML (with varying *TP53* allelic states) as a function of therapeutic intervention (Figure 4). VAFs were adjusted for CNV, as shown in the representative pictogram (Supporting Information Figure S3).

**FIGURE 5:**
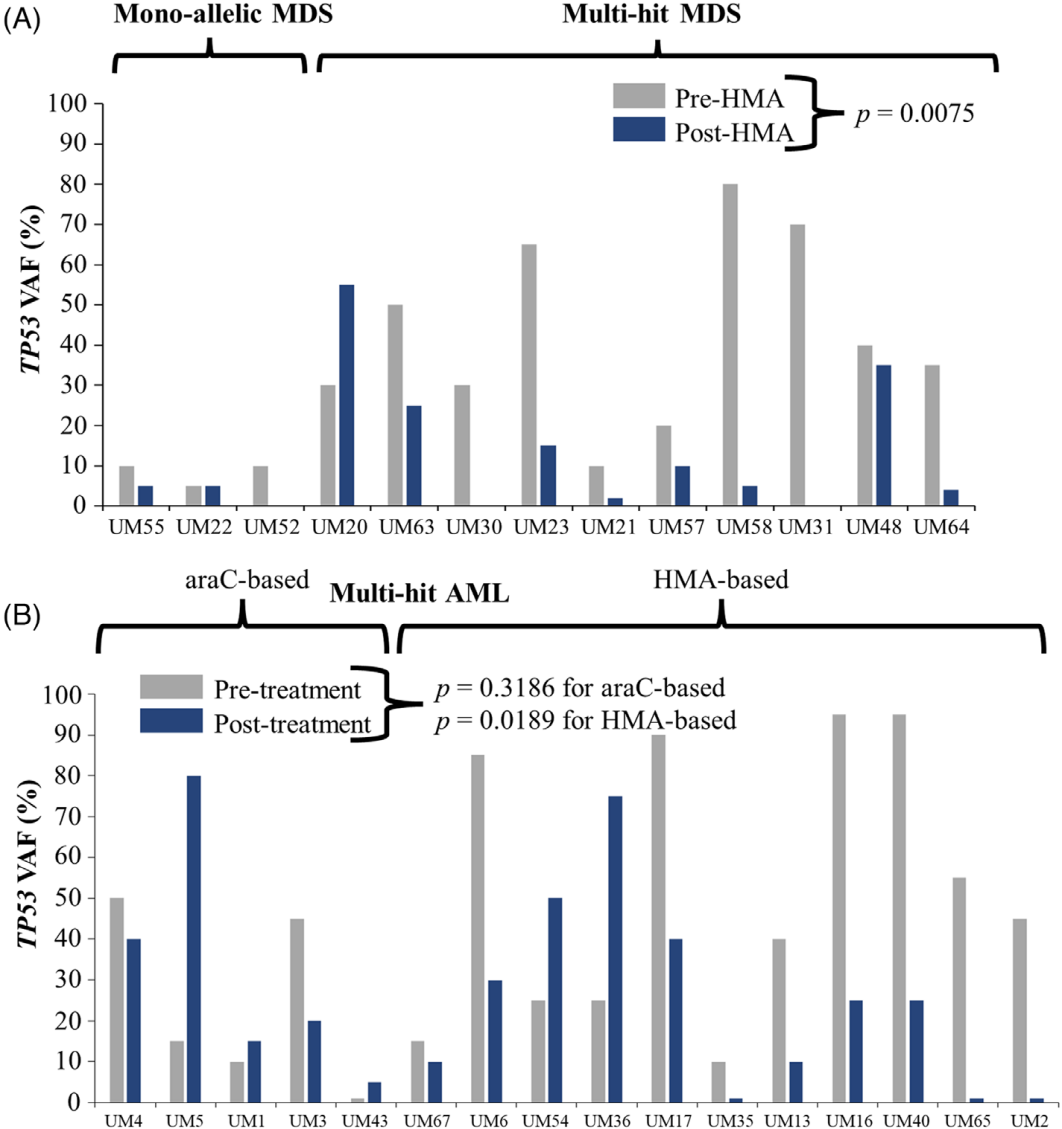
(A) *TP53* VAF before and after therapy for patients with monoallelic and multihit *TP53*‐aberrant MDS. (B) *TP53* VAF before and after therapy for patients with multihit *TP53*‐aberrant AML. Paired *t*‐tests for VAFs were performed.

For patients with *TP53*‐aberrant MDS, 17 patients underwent serial bone marrow biopsies during treatment. Of these, 11 (64.7%) had multihit status (Figure 4, *yellow backdrop*). Eight patients (47.1%) underwent HCT, and 7 of these 8 patients had received HMA‐based front‐line therapy (Figure 4, *green backdrop*). For patients with MDS, we assessed the effect of HMA‐based treatment on *TP53* VAF. The mean *TP53* VAF (sum of VAFs) was significantly lower after HMA‐based treatment (35 vs. 12.6%) (*p* = 0.0075) (Figure 5A).

For patients with *TP53*‐aberrant AML, 19 patients underwent serial bone marrow biopsies during treatment. Of these, 14 (73.7%) had multihit status (Figure 4, *yellow backdrop*). Only two patients (10.5%) underwent HCT (Figure 4, *green backdrop*). Five patients (26.3%) received araC‐based induction, while 11 patients (57.9%) initially received HMA‐based induction. Notably, 100% of araC‐based therapy recipients eventually went on to receive HMA later in their treatment course. Only one patient with multihit *TP53* underwent HCT, and this patient had previously received HMA plus venetoclax. For patients with AML with multihit *TP53*, we assessed the effect of HMA‐ versus araC‐based front‐line therapy on *TP53* VAF. HMA therapy led to a mean fold reduction in *TP53* VAF (pre‐ to post‐treatment) of 11.7 ± 5.8 (*p* = 0.0189), while araC‐based therapy actually led to a mean fold increase (but not statistically significant) in *TP53* VAF (pre‐ to post‐treatment) of 2.6 ± 1.05 (*p* = 0.3186) (Figure 5B). HMA‐based treatment resulted in a significantly greater reduction in the VAF in aberrant clones and subclones compared to araC‐based treatment. Some patients were started on first‐line therapy after diagnosis but were unable to proceed with serial bone marrow biopsies, mostly due to death prior to response assessment (Supporting Information Figure S4).

### 3.6 Clinical fate and post‐transplant outcomes

**FIGURE 6:**
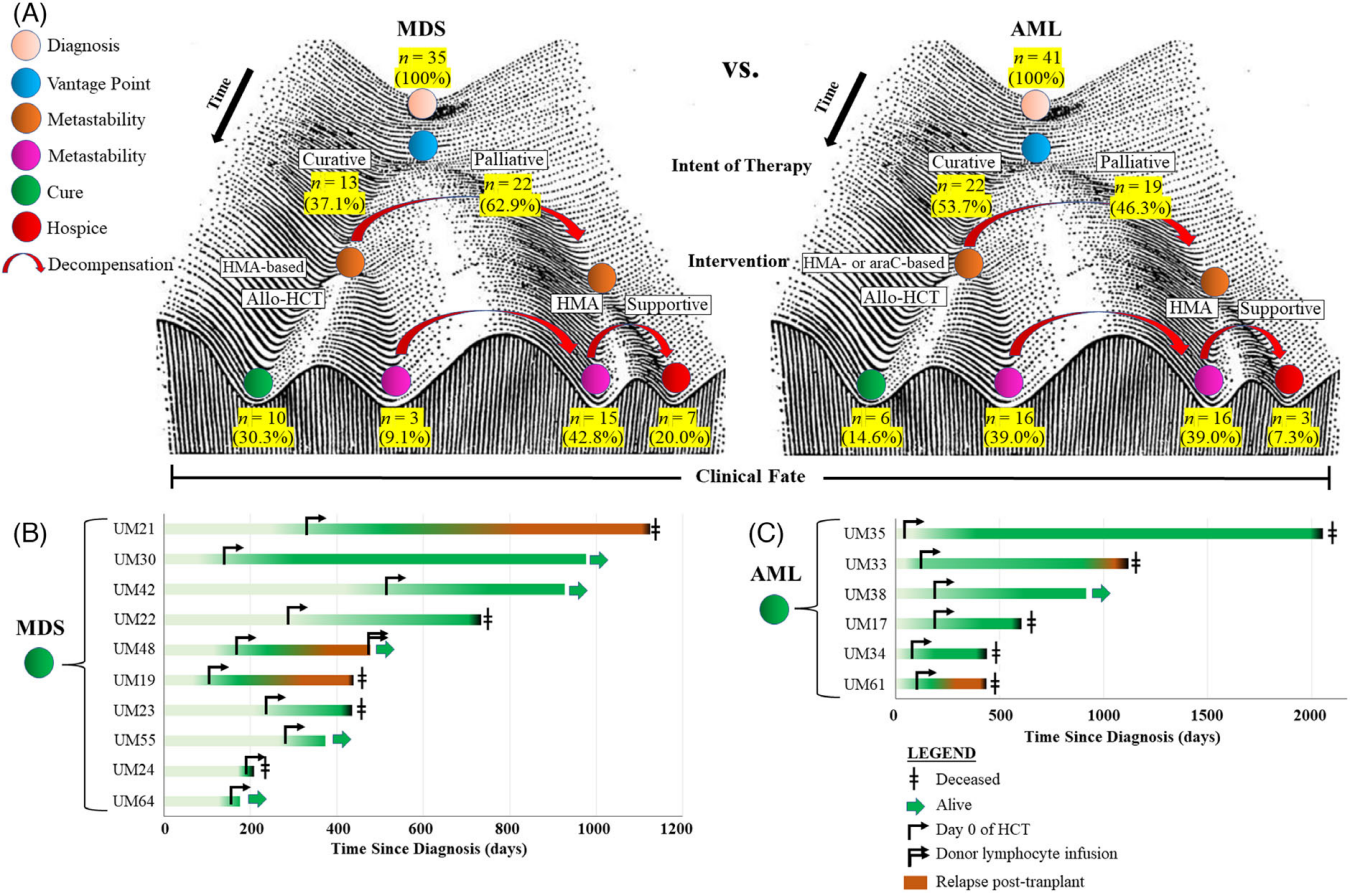
(A) Clinical fate specification based on key therapeutic decision points for patients with *TP53*‐aberrant MDS (*left*) versus AML (*right*). Fate specification was adapted from Waddington's epigenetic landscape. (B) Natural history of patients with MDS after HCT (*n* = 10). (C) Natural history of patients with AML after HCT (*n* = 6).

We assessed the clinical trajectory of patients with *TP53*‐aberrant MDS versus AML based on key management decision points including decisions about curative versus palliative intent therapy and candidacy for HCT (Figure 6A). Compared to AML, most patients with MDS (62.9%) proceeded with palliative intent therapy at the time of diagnosis, largely due to their older age and suboptimal performance status at the time of diagnosis. Most patients with AML (53.7%) proceeded initially with curative intent, in contrast to MDS. However, only 14.6% of patients with AML eventually proceeded with HCT (despite curative intention at diagnosis), largely due to clinical decompensation and partly due to disease progression during front‐line therapy. Among HCT recipients for MDS (*n* = 10), 7 (70%) remained in remission (Figure 6B). The best long‐term outcomes were seen in patients with low *TP53* VAF. Among HCT recipients for AML (n = 6), 4 (66.7%) remained in a morphologic leukemia‐free state (remission) (Figure 6C). Median OS was 734 days for transplant recipients.

## 4 DISCUSSION

To date, multiple studies have shown the impact of *TP53* mutations on survival, though most studies evaluated this genetically defined subset as a homogeneous group, in comparison to other mutation‐specific groups [2, 14, 15]. Bernard et al. showed survival implications for multihit *TP53*‐mutant MDS, which we were unable to reproduce perhaps due to our smaller sample size [2]. In our current study, we noted significant heterogeneity with respect to genomic states and outcomes for patients with *TP53* disruptions. Although patients with MDS and AML shared common gross structural aberrations and intragenic *TP53* aberrations (Figure 1), there was substantial diversity in each patient's allelic state, response to front‐line therapy, HCT candidacy, and ultimately clinical outcomes. Our data enhances upon prognostic discrimination within this heterogeneous group of patients; such heterogeneity within this population was mostly a function of the genomic state of *TP53* for each patient, with less impact of morphologic state or clinical disease label. Our data suggests that the boundary between classification as MDS or AML remains indistinct, but a functional and clinically relevant distinction is observed with varying *TP53* genomic status. Importantly, among all patients with AML (single‐hit or multihit), only one patient remained alive at the longest time to follow‐up (∼5.5 years).

Our data are consistent with findings by Grob et al. showing no significant differences in molecular characteristics between MDS with excess blasts and AML [11]. Regarding survival, Grob et al. suggested that AML and MDS with excess blasts do not differ with respect to survival [11]. In contrast, our findings suggest significant prognostic heterogeneity between MDS and AML when patients are stratified based on allelic state and VAF. Within the multihit category, we found that the most inferior outcomes are seen in patients with AML with VAF >50%, which confers obligatory biallelic disruption in at least some percentage of cells. Early determination of allelic state and VAF may also guide optimal front‐line therapeutics, especially when treatment is given with curative intent.

There is limited literature on clonal and subclonal diversity in a heterogeneous group of patients with *TP53* aberrations. Such clonal considerations are important from the standpoint of therapeutics, as certain interventions may only eliminate a fraction of cells within a given patient. We identified a distinct clonal architecture within each patient, and no two patients had the same disease features. In our cohort, HMA‐ and araC‐based regimens had differential effects on various subclones (Figure 4). HMA was more effective in reducing the mutant *TP53* burden compared to cytotoxic induction therapy, and more patients were able to proceed with HCT if they received first‐line HMA‐based therapy. In fact, araC‐based therapy increased the *TP53* VAF, which was likely due to selection pressure in favor of *TP53*‐mutant cells. We did not observe improved OS with the addition of venetoclax (*data not shown*): this may be related to a higher risk for neutropenia and subsequent infections with venetoclax, which can ultimately impair HCT candidacy (Figure 6). This is consistent with prior reports on the outcomes of *TP53*‐mutant myeloid neoplasms after treatment with venetoclax [16]. Prospective trials may be needed to identify optimal front‐line regimens that lead to the best long‐term outcomes.

We did not observe progression from monoallelic to multihit state for any patients: the contributing reasons were that patients died (thus no subsequent bone marrow aspirate showed biallelic state), and patients received treatment (HMA) which blocked the progression and reduced the *TP53* VAF. For example, if a patient had a single mutation (monoallelic) and received HMA, the VAF decreased. If hypothetically there was a group of monoallelic patients who were untreated but lived long enough to follow with serial bone marrow aspirates, then we might capture them converting into biallelic status. For the patients with multihit status, all were biallelic at a time of diagnosis. We can presume that all multihit patients started out as monoallelic but were not captured and diagnosed as monoallelic. The ideal in vitro study to address clonal dynamics is to establish a fresh set of monoallelic hit cells and then follow those cells longitudinally to assess for conversion to biallelic hit. This can also be tested in patient‐derived xenografts.

One limitation to our study is the lack of single‐cell resolution [12]. Our data infers clonal composition from VAFs, CNV, and cytogenetics [17]. The presumption in our study is that if VAF is above 50%, then at least a fraction of the cells has obligatory biallelic disruption. For example, if VAF was 51%, then we can guarantee that at least one cell in that bone marrow has a biallelic disruption (there might be more than one cell with a biallelic disruption, but there is at least one). If VAF is 50% or less, then there are two possible scenarios: (A) every cell has monoallelic disruption at most (none are biallelic), or (B) some cells have biallelic disruption and some have monoallelic disruption.

Another limitation of our study is the varying time to clinical presentation for each patient: the disease likely developed months to years prior to clinical detection, and this can skew the analysis from these diagnostic bone marrow specimens. Lead‐time bias may be present for patients with more indolent disease courses (such as patients with monoallelic states and low *TP53* VAF).

A crucial question continues to revolve around curative intent therapy for this heterogeneous subset, as long‐term survival is seen more often in HCT recipients compared to non‐HCT recipients. Long‐term survival may depend on HCT candidacy from the time of diagnosis, though one's performance status is not static and may depend on the type of front‐line therapy received. Since HCT is a time‐dependent covariate and subject to selection bias, we recognize that our data may not objectively reflect a favorable effect of HCT as an independent variable, as patients who were selected for HCT were likely healthier at baseline than those who were not selected for HCT. Our data supports the use of HMA‐based therapy as front‐line therapy to help reduce the mutant *TP53* allele burden, followed by HCT for patients who remain candidates for HCT. HCT affords durable remission lasting beyond 1 year in most cases (Figure 6B, C). HCT also appears to be the most effective intervention to lead to MRD negativity. Furthermore, we acknowledge another limitation to our study: it is difficult to mix survival studies between patients treated or untreated, as well as those who are more or less intensively treated, or even those who have received an allograft.

Translational efforts have focused on targeting *TP53*‐mutant cells; such agents include the p53 reactivator eprenetapopt (APR‐246), the nutlin analogs, magrolimab, and sabatolimab [18, 19, 20, 21, 22, 23, 24]. However, there are no Food & Drug Administration (FDA)‐approved medications to date, and there are no consensus guidelines for definitive management of *TP53*‐mutant MDS and AML [25]. Recent clinical trials of *TP53* mutation‐directed therapy have not met primary endpoints in Phase III settings [18]. Single‐center studies have shown that HMA with or without venetoclax has some efficacy and may serve as a bridge to HCT, though the median OS is still very short [3, 6, 16, 26]. We note the enrichment of *TP53* mutations in non‐Hispanic White patients, and racial disparities in therapeutic approaches are also worthy of consideration [27]. Further risk stratification using details of the *TP53* genomic state in conjunction with morphologic assessment may guide tailored therapeutics and personalized treatment plans for this heterogeneous group [28, 29, 30, 31, 32, 33, 34].

## AUTHOR CONTRIBUTIONS

SAP and JMG conceived the study. SAP, WKG, and BT wrote the manuscript. JC, AGS, MR, SS, AH, LH, SK, XM, PM, WS, BW, and JMG contributed to data acquisition. SAP, JC, AE, JF, BB, KS, and JMG contributed to critical analysis and revisions. SAP and JMG gave final approval.

## CONFLICT OF INTEREST STATEMENT

SAP served on the COMMANDS advisory board and the Acute Myeloid Leukemia advisory board for Bristol Myers Squibb and served on the Multiple Myeloma advisory board for Pfizer. JC served on advisory boards for Jazz Pharmaceuticals, Bristol Myters Squibb, MERIT CRO, Pfizer, and Amgen; served as a Data Safety Monitoring Board Member with ICON‐AlloVir and ICON‐Prolacta; owns stock in Actinium Pharmaceuticals, Bluebird Bio/2Seventy, Cellectar Sciences, Dynavax Technologies, aTyr Pharma, Gamida Cell Ltd, Novavax Inc, Ovid Therapeutics, Sorrento Therapeutics, TG Therapeutics, Vaxart, and Veru. JMG serves on the Advisory Board for Novartis and holds US Patent No. 9 012 215, US Patent No. 10 222 376, and US Patent No. 11 209 435.

## FUNDING INFORMATION

SAP receives research funding from the UMass Center for Clinical and Translational Science (CCTS) Pilot Project Program grant (NIH/NCATS Grant UL1TR001453). AE was supported by the National Cancer Institute (NCI) award F32CA250304, the Advanced Residency Training Program at Stanford, and the American Society of Hematology Scholar Award.

## ETHICS STATEMENT

Approval was obtained through the UMass Institutional Review Board (IRB) via protocols H00019393 (“AML Database”) and H00013131 (“Blood & Marrow Banking”; NCT01174615).

## PATIENT CONSENT STATEMENT

Approval was obtained through the UMass Institutional Review Board (IRB) via protocols H00019393 (“AML Database”) and H00013131 (“Blood & Marrow Banking”; NCT01174615).

## CLINICAL TRIAL REGISTRATION

The authors have confirmed clinical trial registration is not needed for this submission.

## Supporting information


**FIGURE S1**. OS for all 76 patients with *TP53* aberrations **(A)**. OS for subgroups after stratification based on disease label **(B)**, karyotype **(C)**, and history of chemotherapy exposure **(D)**. Log‐rank *p* values are shown.

**FIGURE S2. (A)** OS as a function of *TP53* VAF, stratified by complex karyotype (*blue*) versus noncomplex karyotype (*pink*). **(B)** OS as a function of age at diagnosis, stratified by complex karyotype (*blue*) versus noncomplex karyotype (*pink*). Coefficients of correlation are shown.

**FIGURE S3**. Representative pictogram of CNV analysis for a patient. Green probe dots represent significant gains. Red probe dots represent significant deletions. Total coverage for sample and control is shown.

**FIGURE S4**. Clonal landscape for patients with only one bone marrow biopsy (diagnostic sample) available. These patients were not able to proceed with subsequent bone marrow biopsies due to death or lack of follow‐up.

Supporting Information

Supporting Information

## Data Availability

The data that support the findings of this study are available in Supporting Information of this article.
