# Supplementary figures and images for: Prognostic heterogeneity and clonal dynamics within distinct subgroups of myelodysplastic syndrome and acute myeloid leukemia with TP53 disruptions
Source: EJHaem. 2023 Sep 11;4(4):1059–70. doi: 10.1002/jha2.791 (PMC10660125; doi:10.1002/jha2.791)

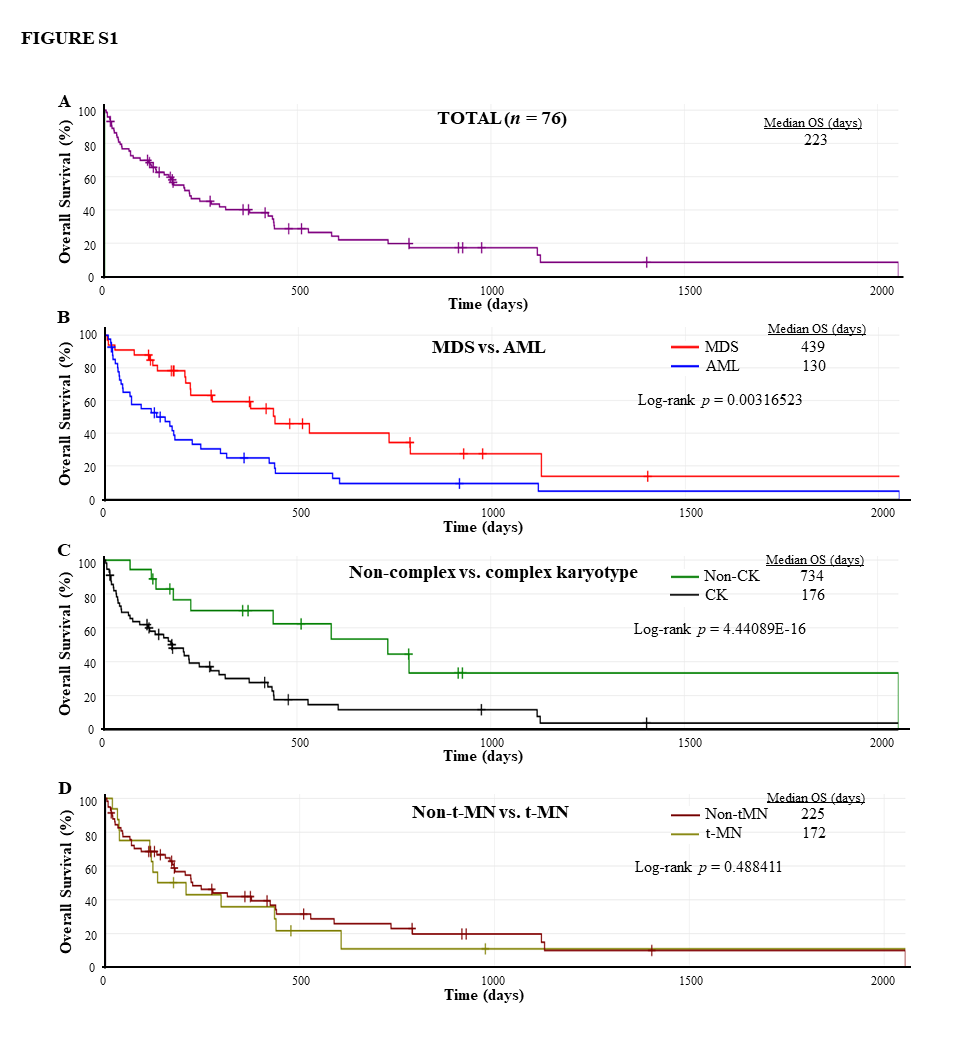

Supplement: Supplementary file 1 — FIGURE S1. OS for all 76 patients with TP53 aberrations (A). OS for subgroups after stratification based on disease label (B), karyotype (C), and history of chemotherapy exposure (D). Log‐rank p values are shown. [file JHA2-4-1059-s003.tif]

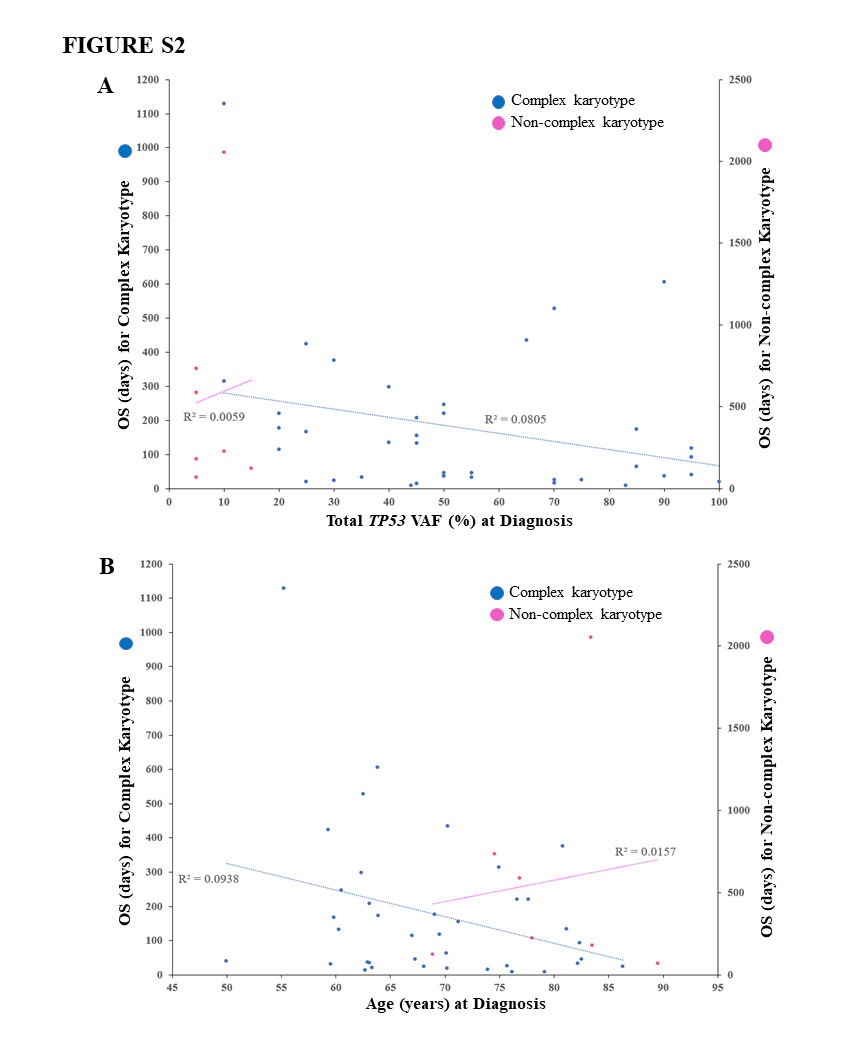

Supplement: Supplementary file 2 — FIGURE S2. (A) OS as a function of TP53 VAF, stratified by complex karyotype (blue) versus noncomplex karyotype (pink). (B) OS as a function of age at diagnosis, stratified by complex karyotype (blue) versus noncomplex karyotype (pink). Coefficients of correlation are shown. [file JHA2-4-1059-s002.tif]

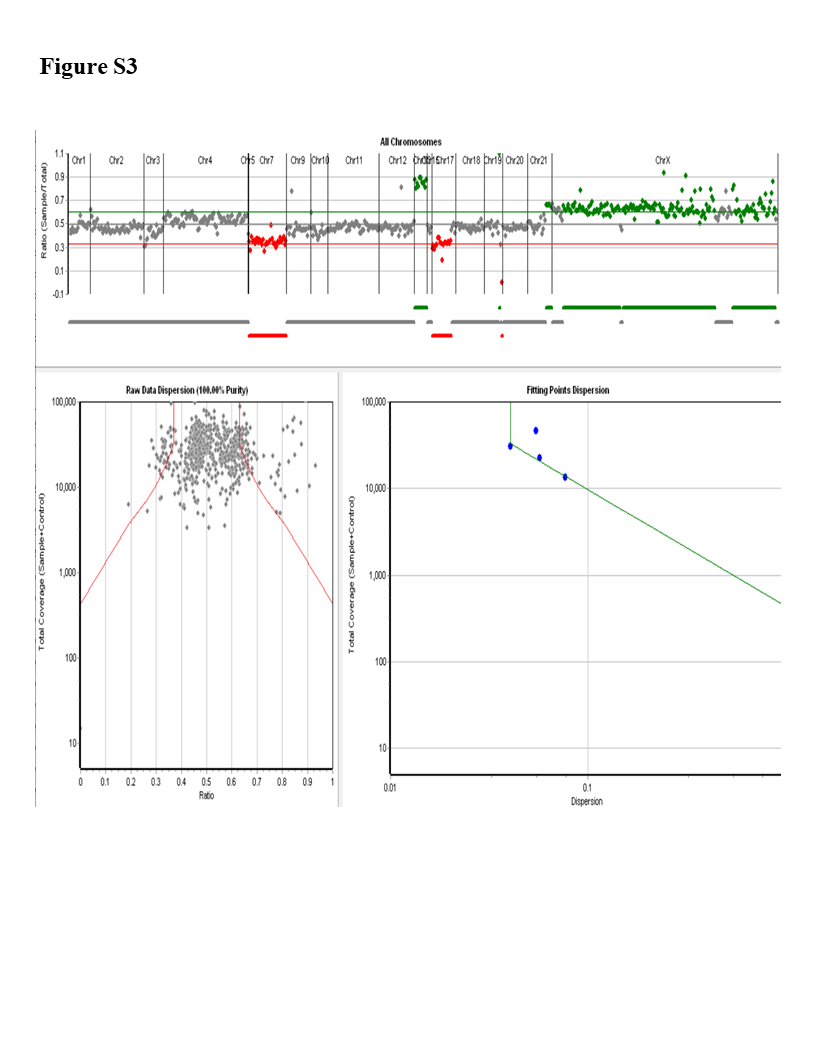

Supplement: Supplementary file 3 — FIGURE S3. Representative pictogram of CNV analysis for a patient. Green probe dots represent significant gains. Red probe dots represent significant deletions. Total coverage for sample and control is shown. [file JHA2-4-1059-s001.tif]

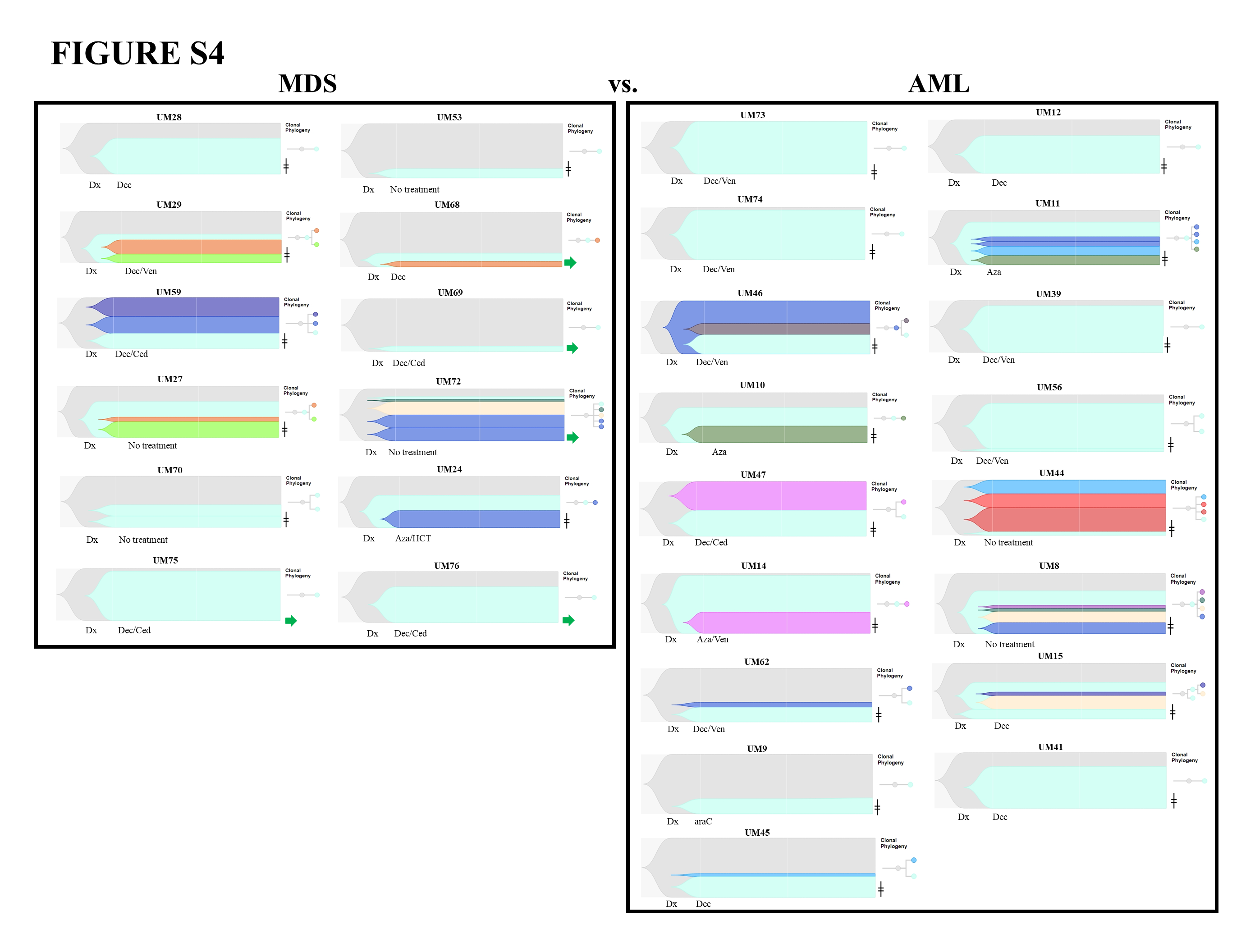

Supplement: Supplementary file 4 — FIGURE S4. Clonal landscape for patients with only one bone marrow biopsy (diagnostic sample) available. These patients were not able to proceed with subsequent bone marrow biopsies due to death or lack of follow‐up. [file JHA2-4-1059-s004.tif]
