# Supplementary material for: Prognostic heterogeneity and clonal dynamics within distinct subgroups of myelodysplastic syndrome and acute myeloid leukemia with TP53 disruptions
Source: EJHaem. 2023 Sep 11;4(4):1059–70. doi: 10.1002/jha2.791 (PMC10660125; doi:10.1002/jha2.791)
Supplement: Supplementary file 5 — Supporting Information [file JHA2-4-1059-s005.docx]

**Supplemental Table S1.** ***TP53* Gene Sequencing Coverage**

| **Gene** | **Coverage** | **Exon** | **Codons Covered** | **Start** | **End** | **Amplicon Size** | **Coverage Depth (250X)** | **Hotspot Coverage?** |
| --- | --- | --- | --- | --- | --- | --- | --- | --- |
| TP53_1_1.165323 | Entire CDS | 10 | 367-393 | 7572852 | 7573032 | 180 | 1000 X |  |
| v3_TP53_1_1.140683 | Entire CDS | 10 | 367-380 | 7572967 | 7573183 | 216 | 2500 X |  |
| TP53_3_1.11586 | Entire CDS | 9 | 332-367 | 7573868 | 7574087 | 219 | 3000 X | yes |
| TP53_5_1.166752 | Entire CDS | Intron |  | 7576494 | 7576706 | 212 |  |  |
| TP53_7_1.120850 | Entire CDS | 8 | 307-331 | 7576783 | 7576945 | 162 | 4000 X | yes |
| TP53_10_1.12998 | Entire CDS | 7 | 261-307 | 7576959 | 7577165 | 206 | 6000 X | yes |
| TP53_10_1.308118 | Entire CDS | 7 | 261-307 | 7576983 | 7577197 | 214 | 6000 X | yes |
| TP53_11_1.234166 | Entire CDS | 6 | 225-260 | 7577393 | 7577620 | 227 | 6500 X | yes |
| TP53_11_1.109858 | Entire CDS | 6 | 225-260 | 7577486 | 7577664 | 178 | 6500 X | yes |
| TP53_13_1.140846 | Entire CDS | 5 | 187-224 | 7578126 | 7578362 | 236 | 2500 X | yes |
| TP53_15_1.90240 | Entire CDS | 4 | 138-187 | 7578309 | 7578518 | 209 | 1500 X | yes |
| v3_TP53_16_1.237842 | Entire CDS | 4 | 126-162 | 7578443 | 7578595 | 152 | 1000 X | yes |
| TP53_20_1.164217 | Entire CDS | 3 | 62-125 | 7579279 | 7579499 | 220 | 2000 X | yes |
| TP53_20_1.86000 | Entire CDS | 3 | 45-121 | 7579324 | 7579551 | 227 | 2500 X | yes |
| TP53_22_1.177842 | Entire CDS | 2,3 | 25-49 | 7579538 | 7579730 | 192 | 1000 X | yes |
| v3_TP53_24_1.86538 | Entire CDS | 1 | 10-28 | 7579711 | 7579879 | 168 | 500 X |  |
| v3_TP53_25_1.111200 | Entire CDS | 1 | 1-22 | 7579842 | 7579960 | 118 | 3000 X |  |
