# Supplementary material for: Prognostic heterogeneity and clonal dynamics within distinct subgroups of myelodysplastic syndrome and acute myeloid leukemia with TP53 disruptions
Source: EJHaem. 2023 Sep 11;4(4):1059–70. doi: 10.1002/jha2.791 (PMC10660125; doi:10.1002/jha2.791)
Supplement: Supplementary file 6 — Supporting Information [file JHA2-4-1059-s006.docx]

| **Supplemental Table S2. Adjusted Model for Survival** | | |  |
| --- | --- | --- | --- |
| Adjusted HRs | HR | 95% CI | p-value |
| AML vs. MDS | 3.30 | (1.5, 7.2) | 0.003 |
| Without HCT vs. with HCT | 4.30 | (1.5, 13) | 0.008 |
| 10-unit change in *TP53* VAF | 1.20 | (1.1, 1.3) | 0.001 |
| 10-year change in age | 1.04 | (0.7, 1.5) | 0.840 |
